# Forced Convection in Wavy Microchannels Porous Media Using TiO_2_ and Al_2_O_3_–Cu Nanoparticles in Water Base Fluids: Numerical Results

**DOI:** 10.3390/mi12060654

**Published:** 2021-06-02

**Authors:** Kholoud Maher Elsafy, Mohamad Ziad Saghir

**Affiliations:** Department of Mechanical and Industrial Engineering, Ryerson University, Toronto, ON M5B 2K3, Canada; kelsafy@ryerson.ca

**Keywords:** porous cavity, wavy channels, nanofluids, forced convection, heat enhancement, pressure drop, mesh model

## Abstract

In the present work, an attempt is made to investigate the performance of three fluids with forced convection in a wavy channel. The fluids are water, a nanofluid of 1% TiO_2_ in a water solution and a hybrid fluid which consists of 1% Al_2_O_3_–Cu nanoparticles in a water solution. The wavy channel has a porous insert with a permeability of 10 PPI, 20 PPI and 40 PPI, respectively. Since Reynolds number is less than 1000, the flow is assumed laminar, Newtonian and steady state. Results revealed that wavy channel provides a better heat enhancement than a straight channel of the same dimension. Porous material increases heat extraction at the expenses of the pressure drop. The nanofluid of 1% TiO_2_ in water provided the highest performance evaluation criteria.

## 1. Introduction

Thermal engineering is one of the major fields in which energy conservation and sustainable development demands are increasing requiring more research efforts towards more energy efficient equipment and processes. The petroleum and process industries helped in many ways through the years to enhance the efficiency and find promising solutions [1]. A new era of microelectronic applications and devices has started requiring more thermal management efficiency specially with the obvious increase of the heat flux of chips. Microchannels heat sinks have been investigated firstly by Tuckerman and Pease [2] and a lot after because of the excellent heat transfer efficiency and capacity plus the small dimensions that are needed nowadays in most new technological applications.

Flow and heat transfer from irregular surfaces are often encountered in many engineering applications to enhance heat transfer such as micro-electronic devices, flat-plate solar collectors and flat-plate condensers in refrigerators, geophysical applications, underground cable systems, electric machinery, cooling system of micro-electronic devices, etc. In addition, roughened surfaces could be used in the cooling of electrical and nuclear components where the wall heat flux is known [3]. An extensive amount of research has been performed in the last decades to obtain a better understanding of flow mixing and heat transfer enhancement in channels with geometrical inhomogeneities, such as serpentine channels, asymmetric and symmetric wavy walls, natural convection heat transfer in wavy porous enclosures, wavy microchannels, etc. [3,4,5,6]. In recent years, it has been shown that nanofluids can be applied in heat exchangers to enhance the heat transfer, leading to higher heat exchanger efficiency [7]. 

The advantages of wavy channels are the ease of manufacturing and the significant enhancement of heat transfer as it was operated [8,9]. Direct liquid cooling incorporating microchannels is considered one of the promising solutions to the problem [10,11]. Secondary flow (Dean vortices) is generated when liquid coolant flows through the wavy microchannels. It is found that along the flow direction, the quantity and the location of the vortices may change leading to chaotic advection, which can greatly enhance the convective fluid mixing.

Choi [12] was the first to mention the term nanofluid which refers to any liquid that contains solid metallic particles in submicron scale/nano scale (e.g., Ag, TiO_2_, Cu or Al_2_O_3_). These nanoparticles then are added to water to form a nanofluid which was found to increase the thermal conductivity and enhance the heat transfer performance of the base working fluids [13]. Widely speaking, it was found from different results that the type of nanoparticles used in the base fluid is the main factor to enhance the performance of heat transfer of traditional work fluids such as Ag nanofluid which gave experimentally the higher performance value due to the inherent properties of this metal oxide that overcomes the thermal diffusion that happens due to the small sizes of its particles [14,15]. Lee et al. [16] used the base fluids such as water and ethylene glycol and added nanoparticles of Al_2_O_3_ and CuO to measure the thermal conductivity. The results obtained by them showed that ethylene glycol based nanofluid achieved a higher value of thermal conductivity than the water-based type. Xuan and Roetzel [17] were also studying the performance of heat transfer using nanofluids and had some interesting results. They were able to derive many equations using single and double-phase flow techniques for the analysis of convective heat transfer in nanofluid. The heat transfer enhancement of nanofluids and the laminar flow were investigated by Heris et al. [18,19] in a circle tube, and the results were typical compared to the other research. Flow and heat transfer characteristic of copper-water nanofluid in a two-dimensional channel was studied by Santra et al. [20] using Cu water nanofluid. The results were notable as with the increase of volume fraction of the solid nanoparticles and Reynolds number, the heat transfer rate was found to be higher.

Saghir research team [21,22,23,24,25,26,27,28,29,30,31,32] have experimentally and numerically investigated the forced convection of Al_2_O_3_–Cu hybrid nanofluid, Al_2_O_3_/water nanofluid in porous media at different flow rates and heating conditions in a straight channel. Results revealed that fluid such as water with metallic nanoparticle can enhance the heat extraction by no more than 6% when compared to water circulation. They concentrated their effort in straight channel with identical dimension.

In the present paper, attempt is made to investigate the performance of wavy channel using the same geometrical parameters and heating condition as the straight channel’s cases done by Saghir et al. The novelty of this research is to be able to enhance the heat removal by allowing the flow to circulate a longer pathway when compared to rectangular case. Pressure drop is a main concern for engineering application thus the need to evaluate the Performance Enhancement Criteria. Section 2 presents the problem description, followed by the finite element formulation in Section 3. Section 4 is allocated for the case studied and the conclusion is in Section 5.

## 2. Problem Description

**Figure 1:**
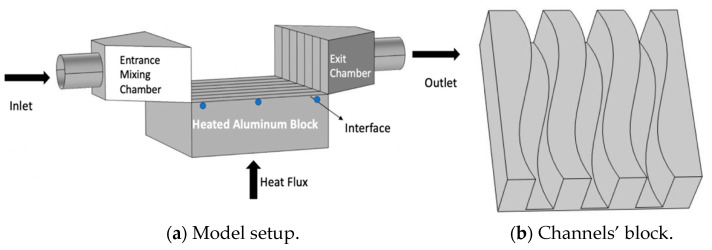
(**a**) Model setup. (**b**) Channels’ block.

In this work, we are investigating a numerical approach of forced convection of nanofluids and hybrid fluid in three-wavy porous channels configuration as shown in Figure 1. The objective is to investigate the heat transfer performance of these fluids that could act as coolants. The three fluids used in the current analysis are water, titanium dioxide/water based nanofluid (1% ${\mathrm{TiO}}_{2}$/water) and aluminum oxide/copper nanoparticle/water-based hybrid fluid (1% Al_2_O_3_–Cu/water). For the hybrid fluid, the single nanoparticles are composed of 90% Al_2_O_3_ and 10% copper [27].

The numerical setup model consists of an inlet tube, a mixing chamber, three wavy porous channels insert, an exit chamber and an outlet tube. The described setup is placed over a heated aluminum block that represents the hot surface. The heated surface of the aluminum block is in direct contact with the bottom of the three channels block. The channel dimensions have a width of 0.00535 m, a height of 0.0127 m and a length of 0.0375 m. The heated aluminum block dimensions are 0.0375 m × 0.0375 m × 0.0127 m. The fluid enters the system with a specific velocity $u_{\mathrm{in}}$ and temperature $T_{\mathrm{in}}$. The temperature was calculated numerically along the fluid path 1mm below the interface of the aluminum block at the centre as shown in Figure 1a. Different flow rates were applied corresponding to 0.05 US gallon per minutes (USGPM), 0.1 USGPM, 0.15 USGPM and 0.2 USGPM. This corresponds to a flow rate of 3.15 × 10^−6^ m^3^/s, 6.3 × 10^−6^ m^3^/s, 9.45 × 10^−6^ m^3^/s and 1.26 × 10^−5^ m^3^/s, respectively. The wavy channels are assumed porous, and the permeabilities used were 10 pore per inches (10 PPI), 20 PPI and 40 PPI. This corresponds to a permeability of 9.557 × 10^−7^ m^2^, 2.38 × 10^−7^ m^2^ and 3.38 × 10^−8^ m^2^, respectively. The porosity is maintained constant at 0.91. The inlet pipe diameter is set equal to 0.01 m, and the heat flux applied to the model, as shown in Figure 1a, has an intensity of 75,000 W/m^2^. Table 1 presents the physical properties of the fluid used in our simulation. The main reason for selecting these fluids is that we have conducted experimental measurement with these fluids in a straight channel configuration, and no sedimentation of the nanoparticles has been observed.

## 3. Governing Equation and Boundary Conditions

In the present work, we attempt to solve the Navier-Stokes equation for the fluid in the entrance and exit chamber combined with the Brinkman formulation for the flow in the porous channels and the energy equation for the fluid in the setup. In addition, the heat conduction equation is solved for the solid surface. The problem is assumed steady state and the flow is in laminar regime. The set of equations used in our model is as follows:

Momentum equations along x, y and z directions, respectively,

(1) $$\rho_{\mathrm{nf}}\left(u\frac{\partial u}{\partial x\;}+v\frac{\partial u}{\partial y}+w\frac{\partial u}{\partial z}\right)=-\frac{\partial p}{\partial x}+{\;\mu }_{\mathrm{nf}}\left(\frac{\partial^{2}u}{\partial x^{2}\;}+\frac{\partial^{2}u}{\partial y^{2}}+\frac{\partial^{2}u}{\partial z^{2}}\right)$$

(2) $$\rho_{\mathrm{nf}}\left(u\frac{\partial v}{\partial x\;}+v\frac{\partial v}{\partial y}+w\frac{\partial v}{\partial z}\right)=-\frac{\partial p}{\partial y}+{\;\mu }_{\mathrm{nf}}\left(\frac{\partial^{2}v}{\partial x^{2}\;}+\frac{\partial^{2}v}{\partial y^{2}}+\frac{\partial^{2}v}{\partial z^{2}}\right)$$

(3) $$\rho_{\mathrm{nf}}\left(u\frac{\partial w}{\partial x\;}+v\frac{\partial w}{\partial y}+w\frac{\partial w}{\partial z}\right)=-\frac{\partial p}{\partial z}+\mu_{\mathrm{nf}}\left(\frac{\partial^{2}w}{\partial x^{2}\;}+\frac{\partial^{2}w}{\partial y^{2}}+\frac{\partial^{2}w}{\partial z^{2}}\right)$$

Continuity equation,

(4) $$\left(\frac{\partial u}{\partial x\;}+\frac{\partial v}{\partial y}+\frac{\partial w}{\partial z}\right)=0$$

Energy conservation equation,

(5) $${\left(\rho \;\mathrm{Cp}\right)}_{\mathrm{nf}}\left(u\frac{\partial T}{\partial x\;}+v\frac{\partial T}{\partial y}+w\frac{\partial T}{\partial z}\right)={\;k}_{\mathrm{nf}}\left(u\frac{\partial^{2}T}{\partial x^{2}\;}+v\frac{\partial^{2}T}{\partial y^{2}}+w\frac{\partial^{2}T}{\partial z^{2}}\right)$$

For the porous flow, the following formulation are used. In particular:

(6) $$\frac{{\mu \;}_{\mathrm{nf}}}{\kappa \;}u=-\frac{\partial p}{\partial x\;}+\mu_{\mathrm{nf}}(\frac{\partial^{2}u}{\partial x^{2}\;}+\frac{\partial^{2}u}{\partial y^{2}}+\frac{\partial^{2}u}{\partial z^{2}})$$

(7) $$\frac{{\mu \;}_{\mathrm{nf}}}{\kappa \;}v=-\frac{\partial p}{\partial y}+\mu_{\mathrm{nf}}\left(\frac{\partial^{2}v}{\partial x^{2}\;}+\frac{\partial^{2}v}{\partial y^{2}}+\frac{\partial^{2}v}{\partial z^{2}}\right)$$

(8) $$\frac{{\mu \;}_{\mathrm{nf}}}{\kappa \;}w=-\frac{\partial p}{\partial z\;}+\mu_{\mathrm{nf}}\left(\frac{\partial^{2}w}{\partial x^{2}\;}+\frac{\partial^{2}w}{\partial y^{2}}+\frac{\partial^{2}w}{\partial z^{2}}\right)$$

Energy formulation for the porous flow,

(9) $${\left(\rho_{\mathrm{nf}}{\mathrm{Cp}}_{\mathrm{nf}}\right)}_{\mathrm{eff}}\left(u\frac{\partial T}{\partial x\;}+v\frac{\partial T}{\partial y}+w\frac{\partial T}{\partial z}\right)={\left(k_{\mathrm{nf}}\right)}_{\mathrm{eff}}\left(u\frac{\partial^{2}T}{\partial x^{2}\;}+v\frac{\partial^{2}T}{\partial y^{2}}+w\frac{\partial^{2}T}{\partial z^{2}}\right)$$

The effective conductivity and the heat capacity combine the porous material and the flow. For further information, readers should consult the reference by Bayomy and Saghir [29]. For the purpose of studying the performance of the wavy channel, two important parameters have been investigated. The first is the local Nusselt number, and the second is the Performance Enhancement criterion. The Nusselt number is defined as the ratio of the convective heat coefficient multiplied by the inlet pipe diameter over the water conductivity (i.e., $\frac{\mathrm{hD}}{k_{w}}$). The heat convection coefficient is known as the ratio of the heat flux over the temperature θ which is the temperature calculated at 1 mm below the interface minus the inlet temperature T_in_ (i.e., θ = T − T_in_). The blue dots shown in Figure 1a indicate the location where the temperature was calculated.

The performance evaluation criterion is an important parameter which combines the average Nusselt number and the friction factor. Nanofluid and hybrid fluid exhibit a larger pressure drop when compared to water. In our analysis, the performance evaluation criteria is shown in Equation (10).

(10) $$\mathrm{PEC}=\frac{\bar{\mathrm{Nu}}}{{\left(f\right)}^{0.333}}$$

Here $\bar{\mathrm{Nu}}$ is the Nusselt number defined earlier, and f is the friction coefficient in the channels.

The fanning friction coefficient is known to be represented by Equation (11).

(11) $$f=\frac{4\left(\Delta p\right)}{\left(\frac{L}{D}\right)\left(\rho_{\mathrm{nf}}\right)\left(u_{\mathrm{in}}^{2}\right)}$$

The pressure difference shown in Equation (11) is the pressure taken at the middle of the inlet mixing chamber to the one at the middle of the exit chamber. Here L is the channel Length equal to 0.0375 m without waviness. 

### Boundary Conditions and Solution Approach

The boundary conditions used in the model consist of applying an inlet velocity u_in_ and an inlet temperature T_in_. The heat flux is applied at the bottom of the heated block, and the remaining external surface is insulated. Figure 1a shows the graphical location of the boundary condition. Porous material is used as an insert inside the channel. At the exit of the flow, a free flow boundary condition is applied. Different approaches exist in COMSOL to tackle the convergence criteria. In this particular model, the default solver used was the segregated method. Details about this approach could be found in any finite element’s textbook. The convergence criterion is clearly explained in COMSOL manual. In a short summary, the convergence criteria were set as follows: at every iteration, the average relative error of u, v, w, p and T were computed. These were obtained using the following relation:

(12) $$R_{c}=\frac{1}{n\;\cdot \;m}\overset{i=m}{\underset{i=1}{\sum }}\overset{j=n}{\underset{j=1}{\sum }}\left|\frac{\left(F_{i,j}^{s+1}-F_{i,j}^{s}\right)}{F_{i,j}^{s+1}}\right|$$

where F represents one of the unknowns, viz., u, v, w, p, or T; s is the iteration number; and (i, j) represents the coordinates on the grid. Convergence is reached if R_c_ for all the unknowns is below 1 × 10^−6^ in two successive iterations. For further information on detailed solution method, the reader is referred for COMSOL software manual [33].

## 4. Mesh Sensitivity Analysis, Convergence Criteria and Model Verification

The mesh sensitivity is examined in purpose of determining the optimal mesh required for the analysis. In the table below, we demonstrated different mesh sensitivity which were investigated following the terminology used by COMSOL software. 

**Figure 2:**
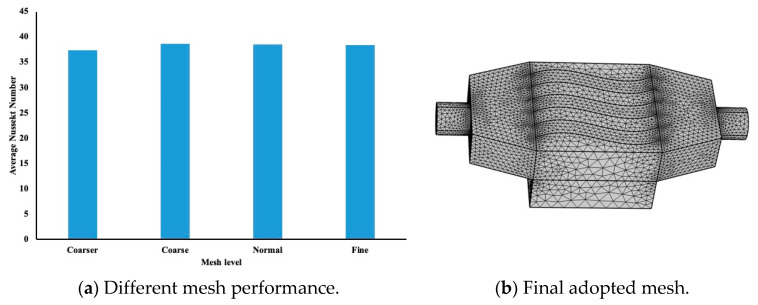
Mesh sensitivity analysis. (**a**) Different mesh performance. (**b**) Final adopted mesh.

The mesh levels that COMSOL supports and the elements number for each mesh level are shown in Table 2. The average Nusselt number was evaluated at 1 mm below the interface in the aluminum block, and the results are represented in the Figure 2a. Here, the heat flux applied is equal to 75,000 W/m^2^, and the conductivity of the water was used to evaluate the Nusselt number. It is evident that a coarse or normal mesh level will be suitable to be used in the COMSOL model. Figure 2b, presents the finite element mesh used in our simulation with normal mesh level. 

The model has been tested against experimental data to demonstrate the model accuracy. Welsford, Saghir et al. [31], Plant and Saghir [24,27] and Delisle, Saghir et al. [32] conducted experimental measurement of heat enhancement in straight porous channel. They used the same proposed numerical model except the channels were straight channels. Identical boundary conditions are used as well. Results revealed a good agreement between the experimental measurement and the numerical code. Thus, the accuracy of the current numerical model.

## 5. Results and Discussion

In the present study, an attempt is made to investigate the effectiveness of wavy channel in improving heat enhancement. Different flow rates were applied corresponding to 0.05 US gallon per minutes (USGPM), 0.1 USGPM, 0.15 USGPM and 0.2 USGPM. This corresponds to a flow rate of Q_1_ = 3.15 × 10^−6^ m^3^/s, Q_2_ = 6.3 × 10^−6^ m^3^/s Q_3_ = 9.45 × 10^−6^ m^3^/s and Q_4_ = 1.26 × 10^−5^ m^3^/s, respectively. The question the authors raised is whether a wavy channel leads to a better performance enhancement criterion when compared to a straight channel? Different fluids are used in the current analysis with water, then a 1% TiO_2_ nanoparticles diluted in 99% water, and finally, a hybrid fluid which consists of 1% nanoparticles containing 90% Al_2_O_3_ and 10% copper diluted in 99% water. The differences between these fluids are their conductivity, density and viscosity. Thermal conductivity may affect the Nusselt number whereas the viscosity, specific heat and density can affect the friction coefficient and thus the pressure drop. Different flow rates will be applied, but the heating condition remains the same with a heat flux of 75,000 W/m^2^. The model was studied for three different permeabilities by maintaining the porosity constant at 0.9.

### 5.1. Heat Enhancement Using Water as Working Fluid

**Figure 3:**
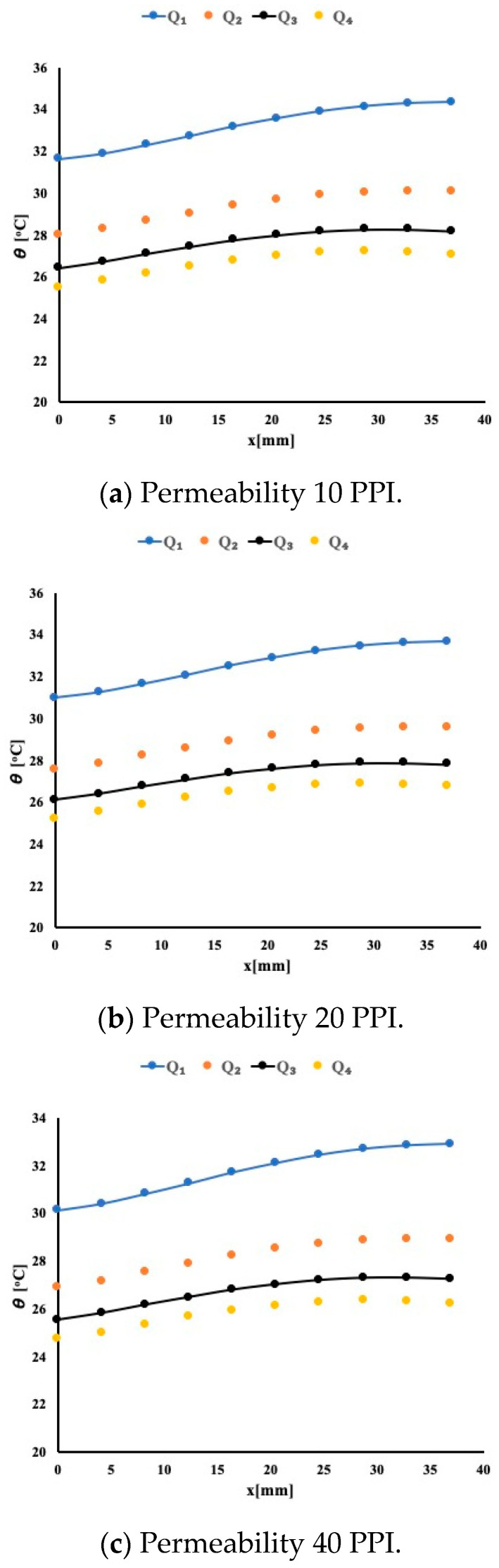
Temperature distribution with water as working fluid.

**Figure 4:**
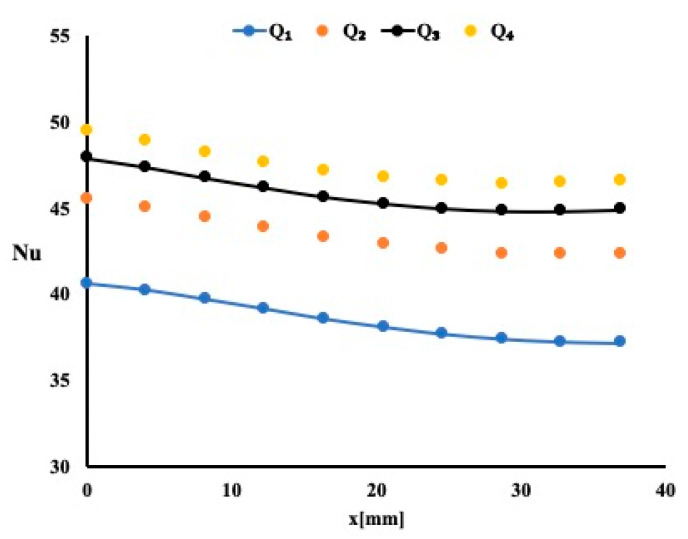
Local Nusselt number variation for different flow rates.

Figure 3 presents the temperature distribution 1 mm below the interface for the three porous material types and for four different flow rates as stated earlier. The temperature profile shows that at the beginning of the flow entrance, the boundary layer is very small, thus allowing the heat to pass from the solid block to the fluid. However, as the boundary layer starts developing, it appears that it reduces the amount of heat extracted from block. This temperature profile is identical regardless of the permeability of the material. By carefully examining the temperature magnitude, it is noticeable that as the permeability varies from 10 PPI to 40 PPI, the heat extraction is improving. This is shown as the temperature in the heated block drop in magnitude. It is evident that having porous material helps to absorb more heat. To study further the heat extraction, Figure 4 presents the local Nusselt number variation for the case or a permeability of 40 PPI.

A negative slope for the local Nusselt number variation is an indication that as the boundary layer developed, the heat extraction decreased accordingly. It is evident for this case that as the flow rate increased, the Nusselt number increased. This increase is noticeable around 3% for the highest flow rate of 0.2 USGPM. For a constant flow rate of 0.2 USGPM, one may notice that the average Nusselt number increases by 3% when compared to the case of 10 PPI and by an additional 2% when compared to 20 PPI case. This is another indication that as the permeability increases the heat extraction improves. Similar observations are noted for three remaining flow rates.

### 5.2. Heat Enhancement Using 1% TiO_2_ in Water as Working Fluid

**Figure 5:**
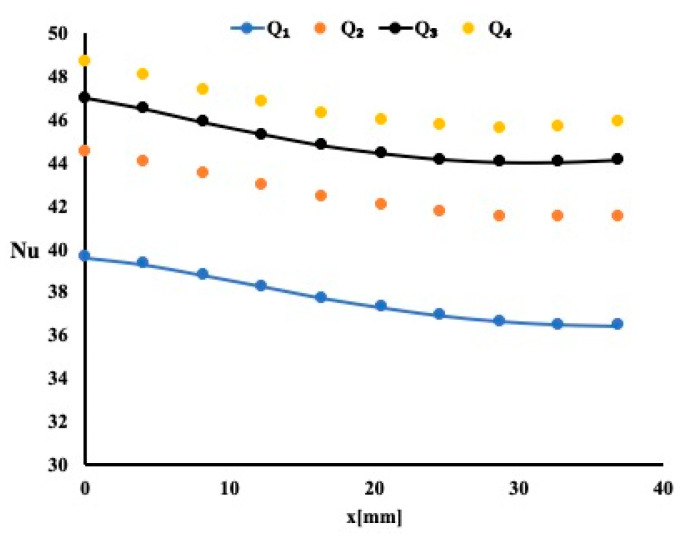
Local Nusselt number variation for a permeability of 20 PPI.

The previous model is repeated by using Titanites as the working fluid. As indicated earlier, a 1% Titanium oxide in water solution is used. The nanoparticles diameter is found to be around 31 nm. Figure 5 presents the local Nusselt number variation along the flow for the four flow rates used and for a permeability of 20 PPI.

Since the thermal conductivity of the Titanite is higher, one expects a heat enhancement improvement when compared to water solution. By carefully examining the average Nusselt number ad for the same permeability, the 1% TiO_2_/water nanofluid exhibits a higher average Nusselt number when compared to water. This improvement is merely 0.5% for a flow rate of 0.2 USGPM. As the permeability increases to 40 PPI, the additional increase in average Nusselt number is found to be around 0.5% as well. Then, one may conclude that nanofluid increases the amount of heat removal.

### 5.3. Heat Enhancement Using 1% (Al_2_O_3_–Cu) in Water as Working Fluid

**Figure 6:**
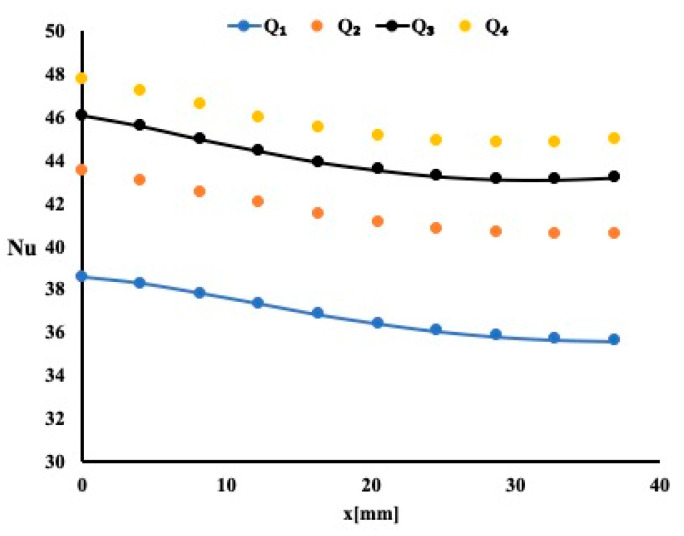
Local Nusselt number variation for a permeability of 10 PPI. (hybrid fluid is the working fluid).

The model was repeated with a hybrid fluid consisting of a single nanoparticle containing Al_2_O_3_ and Copper. Figure 6 displays the local Nusselt number variation for the case of a 10 PPI permeability and for the four flow rates. The Nusselt number variation profile is identical to the other cases, but its magnitude is different.

A similar observation was made regarding the average Nusselt number when compared to water. It is evident that there was not a large change when compared to water. This may be due to the fact that water can provide a good cooling fluid if it is given the chance to circulate more in a hot surface. It is worth noting that using the thermal conductivity of the water in the Nusselt number calculation offers an opportunity to make a correct comparison between the three fluids.

### 5.4. Performance Evaluation Criteria for All Fluids

**Figure 7:**
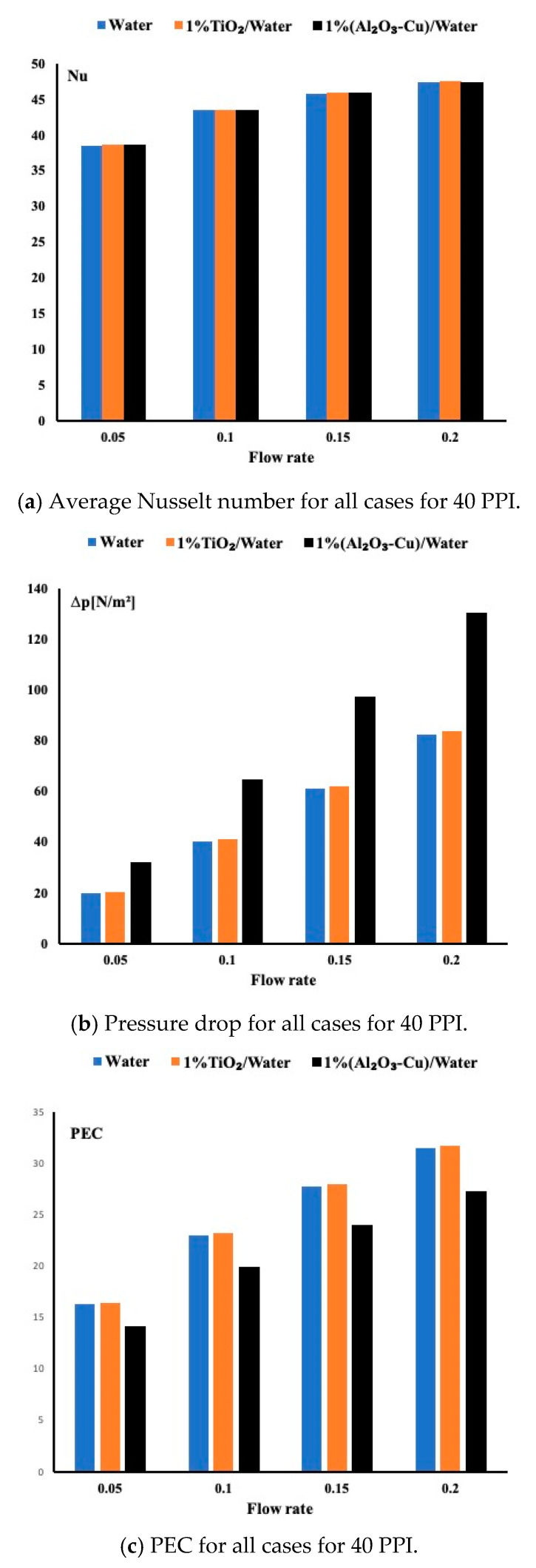
Average Nusselt number, pressure drop and PEC for all cases at a permeability of 40 PPI.

In the previous sections, we have investigated the importance of heat removal based on different flow rates and porous medium permeabilities. However, an important parameter worth investigation is the pressure drop. One may find a suitable fluid for heat removal but at the expense of higher pressure drop. To overcome this issue, it is important to combine the heat effect and the fluid effect by using the performance evaluation criteria. As shown in Equation (10), it is defined as the ratio of the average Nusselt number and the friction factor to the power one third. The friction factor is defined in Equation (11), which combines the physical properties of the fluid, the inlet velocity and the geometrical dimension of the channel. Figure 7 displays the average Nusselt number, the pressure drops and the performance evaluation criteria for all cases when the permeability is set at 40 PPI. Figure 7c displays the PEC and shows that the TiO_2_/water nanofluid exhibits a slightly better performance than water, and the hybrid is the worst candidate between the three studied fluids. In order to determine the reason, Figure 7a displays the average Nusselt number for the three fluids. Almost all of them have a close to identical heat enhancement which may indicate that the waviness of the channel enhances heat removal regardless the fluid used. However, in Figure 7b, a large pressure drop is found for the hybrid fluid contrary to the other remaining two fluids. Thus, there is justification for a higher PEC for the water and the TiO_2_ nanofluid.

**Figure 8:**
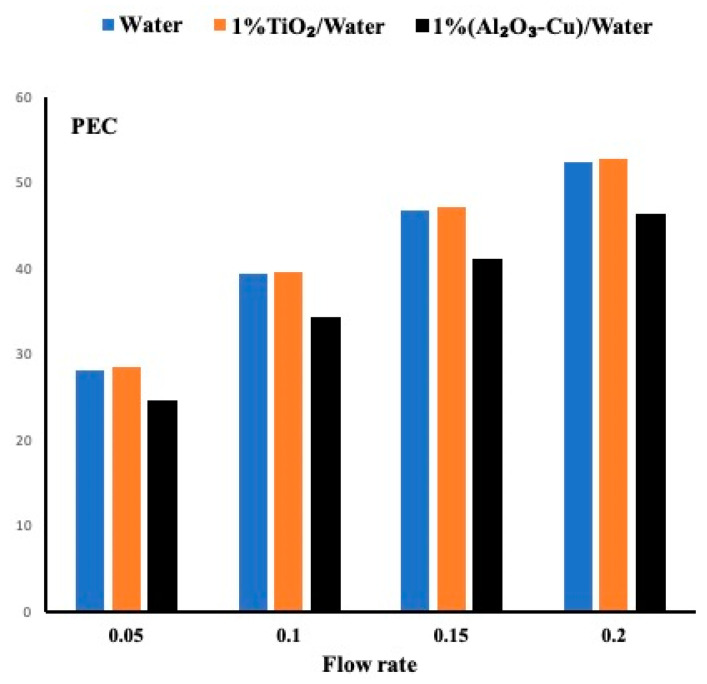
PEC for all cases when the permeability is set at 20 PPI.

As the permeability changes to 20 PPI, the fluid circulates with less obstruction, thus less pressure drop is observed. Figure 8 presents the PEC for all cases at a permeability of 20PPI. It is evident from this figure that the nanofluid slightly outperformed the water and more evident that the hybrid performance is very weak. It is obvious that as the flow rates increase, the PEC increases accordingly.

**Figure 9:**
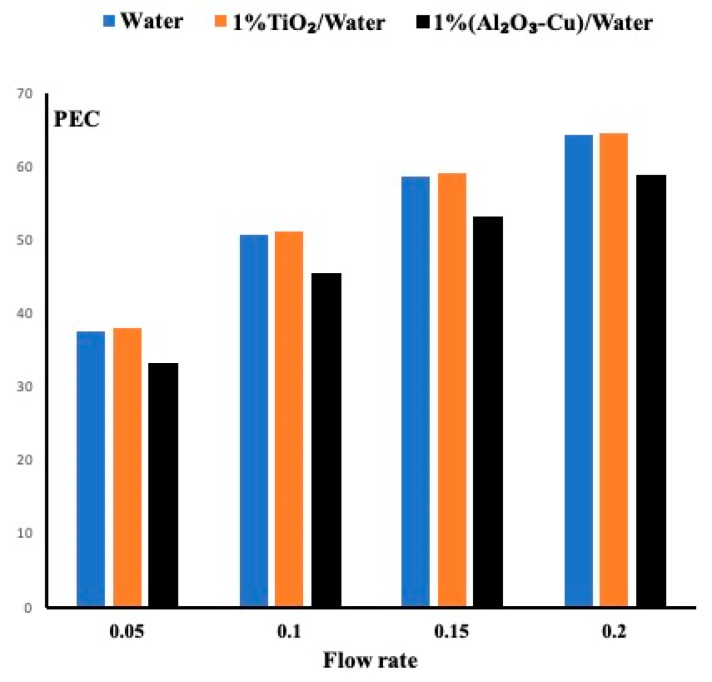
PEC for all cases and for a permeability of 10 PPI.

Finally Figure 9 presents the cases when the permeability is set at 10 PPI. Larger pore provides less pressure drop and thus higher PEC. Nevertheless, the same observation is justified here which indicates that the TiO_2_ nanofluid exhibits the highest performance evaluation criteria followed by the water. The hybrid fluid due to its high pressure drop exhibits the lowest PEC.

## 6. Conclusions

This paper presented a numerical study of the heat performance of three different fluids mainly a single fluid water, a nanofluid 1% ${\mathrm{TiO}}_{2}$/water and a hybrid fluid 1% (${\mathrm{Al}}_{2}O_{3}$–Cu)/water. Three wavy channels with a porous insert, having the three different permeabilities of 10 PPI, 20 PPI and 40 PPI, were investigated numerically. For each case, four different flow rates were implemented. Results reveal the following:

1. Channel waviness allows the flow to circulate longer than a straight channel, thus providing higher heat extraction.
2. By increasing the flow rate, heat enhancement is improved.
3. The presence of porous material helps in heat removal, and as the permeability increases, the pressure drop in the channel decreases accordingly.
4. Amongst the three fluids, the TiO_2_ nanofluid exhibits slightly better performance than the water based on the Performance Evaluation Criteria coefficient. The hybrid fluid provided less performance due to the large pressure drop it exhibited.

## Figures and Tables

**Table 1:** Thermo-physical properties of the fluid used in the analysis [27,28,29].

Fluid	$\mu_{\mathrm{nf}}\;(\mathrm{kg}/m\cdot s)$	$\rho_{\mathrm{nf}}\;(\mathrm{Kg}/m^{3})$	$Cp_{nf}\;(J/\mathrm{Kg}\cdot K)$	$k_{nf}\;(W/m\cdot K)$	Pr (Prandtl Number)
Water	0.001002	998.2	4182	0.613	6.83
1% TiO_2_-0.99 Water	0.001019	1030	4040	0.835	4.93
1% (Al_2_O_3_–Cu)-0.99 Water	0.0016025	1024	4067	0.657	11.11

**Table 2:** Mesh information for different level of meshing [33].

COMSOL Mesh.	Details on Number of Elements at the Boundary and in the Domain
Coarser	71,301 domain elements, 9686 boundary elements, 908 edge elements
Coarse	157,028 domain elements, 17,902 boundary elements, 1276 edge elements
Normal	320,985 domain elements, 29,642 boundary elements, 1674 edge elements
Fine	643,293 domain elements, 47,756 boundary elements, 2140 edge elements

## Data Availability

Not Applicable.

## Nomenclature

$\rho_{\mathrm{nf}}$	Density
μ_nf_	Viscosity
Cp_nf_	Specific heat
k_nf_	Conductivity
κ	Permeability
${\left(\rho_{\mathrm{nf}}{\mathrm{Cp}}_{\mathrm{nf}}\right)}_{\mathrm{eff}}$	Effective heat capacity
(k_nf_)_eff_	Effective conductivity
Δp	Pressure difference
u,v,w	Velocity in x, y and z
p	Pressure
L	Block Length
$\bar{\mathrm{Nu}}$	Average Nusselt number
Nu	Local Nusselt number
f	Fanning friction factor
PEC	Performance Evaluation Criterion
D	Hydraulic diameter
u_in_	Inlet velocity
